# My Best Chum

**Published:** 1912-05

**Authors:** 


### My Best Chum

(*Contributed.*)

I'm the best chum that I ever had; I like to be with me;
I like to sit and tell myself things confidentially.
I often sit and ask me if I shouldn't or I should,
And I find that my advice to me is always pretty good.
I never got acquainted with myself until here of late,
And I find myself a bully chum; I treat me simply great.
I talk with me and walk with me, and show me right and wrong;
I never knew how well myself and me could get along.
I never try to cheat me; I'm as truthful as can be;
No matter what may come or go, I'm on the square with me.
It's great to know yourself and have a pal that's all your own;
To be such company for yourself, you're never left alone.
You'll try to dodge the masses, and you'll find a crowd's a joke,
If you only treat yourself as well as you treat the other folk.
I've made a study of myself, compared with me the lot,
And I've finally concluded I'm the best friend that I've got.
Just get together with yourself, and trust yourself with you,
And you'll be surprised how well yourself will like you if you do.

